# Potential role of blood pressure variability and plasma neurofilament light in the mechanism of comorbidity between Alzheimer's disease and cerebral small vessel disease

**DOI:** 10.1002/alz.14056

**Published:** 2024-06-19

**Authors:** Qin Li, Shu Su, Yuxue Feng, Meng Jia, Jiehong Zhan, Zixuan Liao, Jiayu Li, Xiaofeng Li, for the Alzheimer's Disease Neuroimaging Initiative

**Affiliations:** ^1^ Department of Neurology The Second Affiliated Hospital of Chongqing Medical University Chongqing China; ^2^ Department of Epidemiology and Biostatistics The Second Affiliated Hospital of Chongqing Medical University Chongqing China; ^3^ Department of Neurology University of the Chinese Academy of Sciences Chongqing Renji Hospital Chongqing China; ^4^ Key Laboratory of Major Brain Disease and Aging Research (Ministry of Education) Chongqing Medical University Chongqing China

**Keywords:** Alzheimer's disease, biomarker, blood pressure variability, cerebral small vessel disease, neurofilament light

## Abstract

**INTRODUCTION:**

Long‐term blood pressure variability (BPV) and plasma neurofilament light (pNfL) have been identified as potential biomarkers for Alzheimer's disease (AD) and cerebral small vessel disease (CSVD). However, the relationship between BPV, pNfL, and their association with the comorbidity of AD and CSVD remains unknown.

**METHODS:**

Participants with normal cognition and mild cognitive impairment from the Alzheimer's Disease Neuroimaging Initiative study were included in the data analysis. Linear mixed‐effects regression models and causal mediation analyses were conducted to investigate the relationship among BPV, pNfL, comorbidity‐related brain structural changes (hippocampal atrophy and white matter hyperintensities [WMH]), and cognitive function.

**RESULTS:**

BPV was associated with pNfL, volumes of hippocampus and WMH, and cognition. pNfL mediated the effects of BPV on brain structural changes and cognition.

**DISCUSSION:**

Our findings suggest a potential role of BPV and pNfL in the mechanism of comorbidity between AD and CSVD, underscoring the importance of BPV intervention in the general population.

**Highlights:**

- Individuals with both Alzheimer's disease (AD) and cerebral small vessel disease (CSVD) pathologies had elevated blood pressure variability (BPV) and plasma neurofilament light (pNfL).
- The association between different components of BPV and brain structural changes may vary.
- BPV was associated with pNfL levels independent of average blood pressure.
- pNfL mediated the effects of BPV on comorbidity‐related brain structural changes and cognitive performance.

## 1 BACKGROUND

Alzheimer's disease (AD) and cerebral small vessel disease (CSVD) stand as the major causes of dementia in elderly individuals,^1^, ^2^ and they frequently coexist.^3^, ^4^ The comorbidity of CSVD in AD patients may complicate the treatment of AD, as it requires a comprehensive approach to address the pathologies associated with both AD and CSVD.^5^ Until now, the precise mechanisms underpinning the comorbidity of AD and CSVD remain incompletely elucidated. Currently, it is known that AD and CSVD share some common risk factors, such as elevated long‐term blood pressure variability (BPV),^6^, ^7^ and biomarkers, such as neurofilament light (NfL).^8^, ^9^ The relationship between these risk factors and biomarkers in AD patients comorbid with CSVD remains unclear. A more comprehensive investigation that focuses on AD patients with CSVD and examines those factors may be helpful for uncovering the underlying mechanisms of their comorbidity.

**FIGURE 1 alz14056-fig-0001:**
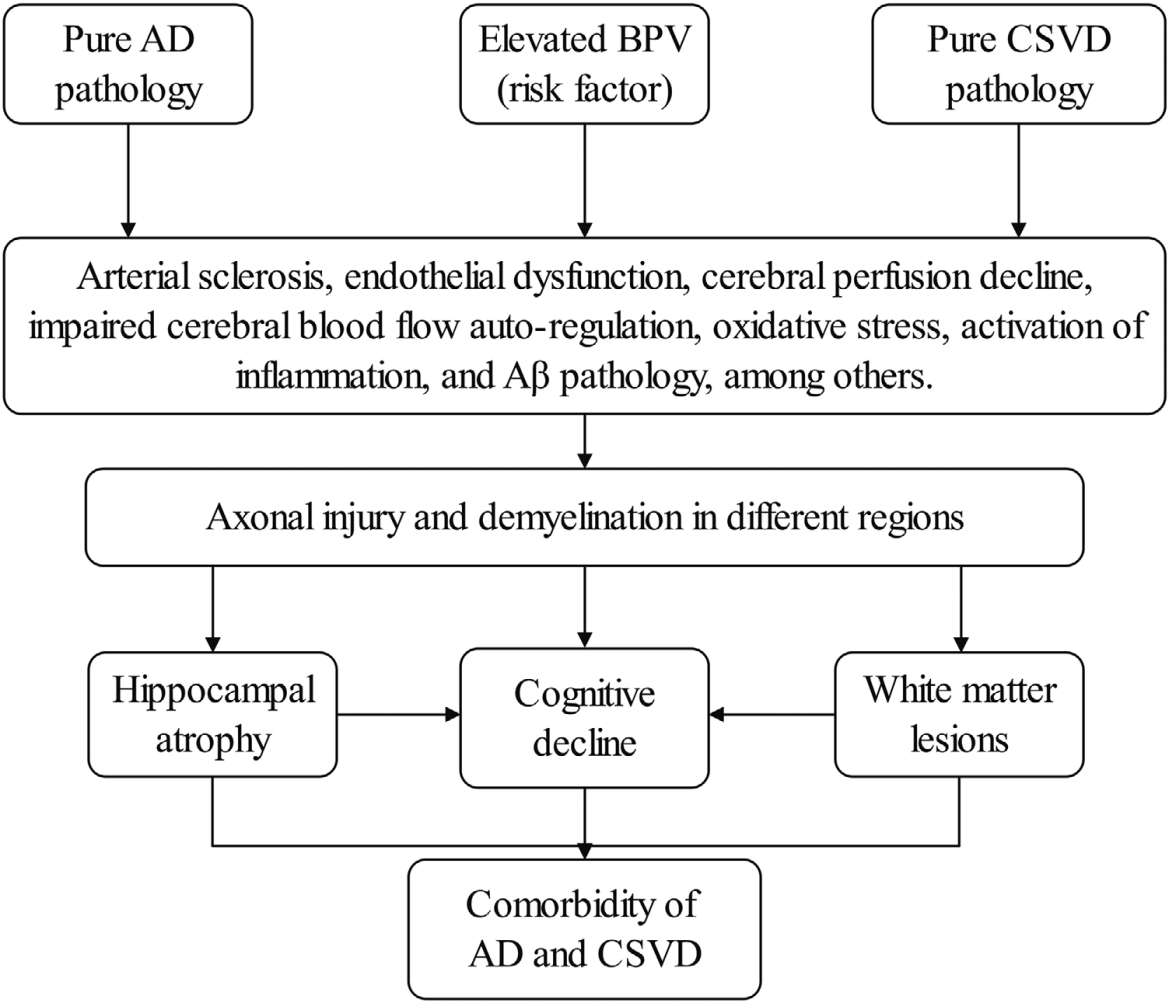
The potential mechanisms by which BPV contributes to the comorbidity of AD and CSVD. Aβ, amyloid beta; AD, Alzheimer's disease; BPV, blood pressure variability; CSVD, cerebral small vessel disease.

Long‐term BPV is an emerging risk factor for AD and CSVD,^6^, ^7^ and has been linked to biomarkers, such as amyloid beta (Aβ) burden, hippocampal atrophy, and white matter hyperintensities (WMHs).^10^, ^11^ These three biomarkers stand for the presence of comorbidity between AD and CSVD.^12^ Furthermore, studies using diffusion tensor imaging (DTI) techniques have demonstrated that elevated BPV is associated with poorer brain white matter integrity.^10^ Because white matter primarily consists of axons,^13^ it suggests that higher BPV may be associated with axonal injury. Concerning AD, there is evidence that axonal injury occurs early and is associated with subsequent neuron death,^14^ while brain structural changes and cognitive impairment occur later.^15^ Therefore, we hypothesize that exposure to higher BPV (risk factor) may lead to micro‐level axonal injury in brain regions that contain axons, such as white matter and hippocampus (which are also rich in neurons).^13^ When these lesions accumulate to a certain extent, they may result in macro‐level changes in brain structural and cognitive decline and thus may be associated with the comorbidity of AD and CSVD (Figure 1).

NFL is a well‐known surrogate marker for axonal injury, degeneration, and loss, with elevated levels in plasma and cerebrospinal fluid (CSF) upon axonal injury.^16^ It shows good consistency with DTI metrics in reflecting the severity of axonal injury.^17^ Herein, plasma neurofilament light (pNfL) has been widely used as a biomarker of axonal injury due to its convenient detection and low cost.^9^ pNfL is found to be elevated in the early stages of AD,^18^ and associated with Aβ burden, hippocampal atrophy, WMH, and cognition.^19^, ^20^, ^21^ In this study, we used hippocampal volume and CSF Aβ42 as representative measures of AD pathology, WMH volume as a representative measure of CSVD pathology, and pNfL levels as representative measures of axonal injury. We investigated the association of BPV with pNfL, comorbidity‐related brain structural changes, and cognition. Considering the current emphasis on prodromal AD as an ideal target for intervention,^22^ our investigation predominantly focused on individuals classified as cognitively normal (CN) and those with mild cognitive impairment (MCI).

## 2 METHODS

### 2.1 Data source

Data were obtained from the Alzheimer's Disease Neuroimaging Initiative (ADNI) database (http://adni.loni.usc.edu), accessed on September 23, 2023. The ADNI cohort was launched in 2003 as part of a public–private partnership, with Principal Investigator Michael W. Weiner, MD, overseeing the project. Approval for the ADNI study was granted by the institutional review boards of all participating institutions. Written informed consent was obtained from all participants or their authorized representatives.

### 2.2 Participants and grouping situation

The comprehensive enrollment process and inclusion criteria for various diagnostic categories within the ADNI cohort have been previously documented.^23^ The inclusion criteria for this study were as follows: (1) age > 55 years; (2) initial diagnosis of CN and MCI with a global Clinical Dementia Rating (CDR) of either 0 or 0.5; (3) a Geriatric Depression Scale (GDS‐15) score < 6; (4) availability of blood pressure (BP) data at baseline, 6 months, 1 year, and 2 years; (5) availability of brain magnetic resonance imaging (MRI), neuropsychological assessments, and pNfL data at baseline, 1 year, and 2 years; (6) baseline availability of AD‐related CSF biomarkers. Exclusion criteria were applied as follows: (1) the usage of any psychoactive medications, (2) a diagnosis of dementia, (3) markedly elevated pNfL levels > 179 pg/mL to exclude conditions like amyotrophic lateral sclerosis and others.^8^

According to the National Institute on Aging and Alzheimer's Association criteria, Aβ positivity can be defined as the AD continuum.^15^ For this study, Aβ positivity was defined as Aβ42 < 977 pg/mL, while Aβ negativity was defined as Aβ42 ≥ 977 pg/mL, aligning with established standards.^24^ Following the criteria established by Cedres et al., a high WMH burden was characterized by WMH volume adjusted total intracranial volume values > 0.00321, while a low WMH burden was defined as adjusted WMH volume ≤ 0.00321.^25^ We use Aβ positivity status to represent the presence of AD pathology and a high WMH burden to signify the presence of CSVD pathology. We compared the differences in BPV and pNfL among different disease groups. The disease groups were defined as follows: Aβ‐negative individuals with low WMH burden were categorized as Aβ−WMH− (controls), Aβ‐negative individuals with high WMH burden were categorized as Aβ−WMH+, Aβ‐positive individuals with low WMH burden were categorized as Aβ+WMH−, and Aβ‐positive individuals with high WMH burden were categorized as Aβ+WMH+, indicating the comorbidity of AD and CSVD.

### 2.3 Neuropsychological assessments

Certified raters conducted the neuropsychological assessments following standardized ADNI protocols (www.adni‐info.org). Various scales were used to evaluate cognition, including CDR, Mini‐Mental State Examination (MMSE), Montreal Cognitive Assessment (MoCA), memory composite score, and executive composite score. Neuropsychological assessment data at baseline, 1 year, and 2 years were obtained from the ADNI file (“MMSE.csv,” “MOCA.csv,” “CDR.csv,” “UWNPSYCHSUM.csv”).

**RESEARCH IN CONTEX**

1. **Systematic review**: Alzheimer's disease (AD) and cerebral small vessel disease (CSVD) often co‐occur in the aging population, yet the underlying mechanisms linking the two remain ambiguous. Studies have revealed associations of long‐term blood pressure variability (BPV) and plasma neurofilament light (pNfL) with AD and CSVD, indicating a potential association between BPV, pNfL, and the comorbidity of AD and CSVD. However, the relationship between BPV, pNfL, and their association with comorbidity remains unexplored.
2. **Interpretation**: Analyses revealed that elevated BPV was associated with pNfL, hippocampal atrophy, white matter hyperintensity progression, and poorer cognition. Moreover, pNfL mediated the effect of BPV on brain structure and cognitive function. These findings provide valuable insights into the potential role of BPV in the mechanisms of comorbidity between AD and CSVD, suggesting that a common risk factor may contribute to the brain pathology associated with both conditions.
3. **Future directions**: It will be necessary to validate the relationship between BPV and pNfL or other axonal injury markers in the prodromal stages of AD. Furthermore, longitudinal tracking and assessment are needed to evaluate the relationship between BPV and the comorbidity of AD and CSVD.

### 2.4 Neuroimaging data

MRI examinations adhered to the ADNI MRI scanning protocol. Four brain tissue (gray matter [including hippocampus], white matter, CSF, and WMH) segmentation methods have been described previously; a thorough description can be found in the ADNI reference documentation “Four Tissue Segmentation in ADNI II.” Brain volume data (including hippocampal and WMH volumes [mL]) at baseline, 1 year, and 2 years were extracted from the ADNI file (“ADNI_UCD_WMH.csv”).[Fig alz14056-fig-0001]

### 2.5 pNfL data and AD‐related CSF biomarkers

pNfL analysis was conducted using the single molecule array (Simoa) technique at the Clinical Neurochemistry Laboratory, University of Gothenburg, Mölndal Campus, Mölndal, Sweden (detailed information available elsewhere: http://adni.loni.usc.edu). CSF samples were collected through lumbar puncture to assess Aβ42, phosphorylated tau (p‐tau)181, and total tau concentrations.^26^ CSF samples were analyzed using the Roche automated immunoassay platform (Cobas e601) and immunoassay reagents at the University of Pennsylvania. The pNfL data at baseline, 1 year, and 2 years were obtained from the ADNI file (“ADNI_BLENNOWPLASMANFLLONG.csv”). AD‐related CSF biomarkers at baseline and 2 years were retrieved from the ADNI file (“UPENNBIOMK_MASTER.csv”).

### 2.6 BP and BPV assessments

Seated brachial artery systolic and diastolic blood pressure measurements were taken from participants at baseline, 6 months, 1 year, and 2 years, following a standardized ADNI protocol described elsewhere (www.adni‐info.org). We calculated the average BP and BPV index, including standard deviation (SD) and variation independent of mean (VIM). VIM is a widely used index for long‐term BPV, computed using the formula VIM = SD/mean^x^,^27^ where x is calculated using a fitting function. The average BP and BPV of systolic blood pressure (SBP), diastolic blood pressure (DBP), pulse pressure (PP), and mean arterial pressure (MAP) were computed. In this study, we mainly used VIM for BPV analysis.

### 2.7 Other assessments and data collection

The following data were gathered from the clinical evaluation file (“ADNIMERGE.csv,” “RECMHIST.csv,” “VITALS.csv”): years of education, current smoking status, height, weight, usage of anti‐dementia and anti‐hypertensive medications, and the presence of vascular risk factors, including hypertension, diabetes mellitus, and atrial fibrillation. The general Framingham Heart Study (FHS) score was calculated to assess vascular risk. The general FHS score comprises a weighted sum of sex, age, total lipoprotein cholesterol, high‐density lipoprotein cholesterol, SBP, treatment for hypertension, current smoking status, and diabetes mellitus status.^28^ Apolipoprotein E (*APOE*) ε4 genotype information was extracted from the ADNI file (“APOERES.csv”). Participants possessing at least one copy of the *APOE* ε4 allele were categorized as *APOE* ε4 carriers.

### 2.8 Statistical analysis

All statistical analyses were conducted using R Programming (version 4.2.2). Statistical significance was set at a two‐tailed *P* < 0.05.

Continuous variables not conforming to a normal distribution underwent transformations before regression analysis. MMSE and MoCA scores were *z* transformed, while WMH volume, pNfL, CSF Aβ42, p‐tau181, and total tau levels were log transformed. One‐way analysis of variance with Bonferroni correction addressed multiple comparisons for normally distributed data. The chi‐square test was used to compare categorical variables, whereas the Kruskal–Wallis with Bonferroni correction was applied to handle multiple comparisons for non–normally distributed continuous variables. The differences in average BP, BPV, baseline biomarkers, and baseline cognitive performance among different groups were compared. As there were variations in age and sex distribution across different groups, a multivariate linear regression analysis was performed to adjust for these factors and calculate the adjusted differences in the indicators among these groups.

To explore the relationship between BPV and pNfL, BPV indexes were categorized into tertiles. The differences in pNfL levels among different BPV tertile groups were compared. *P* values were computed using a multivariate linear model, adjusting for FHS score, *APOE* ε4 status, cognitive status, Aβ status, anti‐hypertensive treatments, and average BP. The longitudinal relationship between BPV and pNfL, adjusted for the aforementioned confounding factors, was assessed using a linear mixed‐effects model. Random effects included participant‐specific intercepts, while fixed effects incorporated the main effects of BPV, with adjustments for previously mentioned confounding variables. The analysis also examined the longitudinal relationship between BPV or pNfL with brain structure (including hippocampal and WMH volumes) and cognitive performance using the same modeling approach.[Table alz14056-tbl-0001]

Causal mediation analyses were used to investigate whether the associations between BPV and brain structural changes and cognition at 2 years were mediated by pNfL. According to the approach proposed by Baron and Kenny,^29^ the following criteria need to be met simultaneously to establish the mediation effect: (1) significant association between BPV and pNfL, (2) significant association between BPV and brain structural changes and cognition, (3) significant association between pNfL and brain structural changes and cognition, (4) weakening of the associations between BPV and brain structural changes and cognition when pNfL (mediator) was included in the regression model. Furthermore, chain mediation analyses were used to investigate whether the association between BPV and cognition at 2 years was mediated by pNfL and brain structural changes. The extent of attenuation or indirect effect was estimated, and significance was determined using 1000 bootstrapped iterations with the “mediate” and “bruceR” package. Each path of the model was adjusted for the aforementioned confounding factors.

## 3 RESULTS

### 3.1 Demographic and clinical characteristics

A total of 413 participants were included in this study, comprising 147 cases of Aβ−WMH− (controls), 64 cases of Aβ−WMH+, 100 cases of Aβ+WMH−, and 102 cases of Aβ+WMH+ (refer to Figure S1 in supporting information for the detailed participant selection procedure). Regarding demographic characteristics (Table 1), the Aβ+WMH+ group exhibited advanced age (mean ± SD, 75.70 ± 5.91 years) compared to both the control group (mean ± SD, 68.83 ± 6.84 years) and Aβ+WMH− group (mean ± SD, 69.88 ± 7.07 years). Furthermore, the Aβ+WMH+ group had a lower percentage of female participants (34.3%) compared to the control group (53.1%).

**TABLE 1 alz14056-tbl-0001:** Baseline characteristics of different groups.

Indexes	Aβ−WMH− (*n* = 147)	Aβ−WMH+ (*n* = 64)	Aβ+WMH− (*n* = 100)	Aβ+WMH+ (*n* = 102)	*P*
**Demographic**					
Age (year)	68.83 (6.84)	75.21 (5.68)^a^	69.88 (7.07)^b^	75.70 (5.91)^[Link]^, ^[Link]^	<0.001
Female (%)	78 (53.1%)	34 (53.1%)	41 (41.0%)	35 (34.3%)^a^	0.013
Education years (year)	16.6 (2.48)	16.3 (2.62)	16.5 (2.62)	16.5 (2.59)	0.874
**Risk factors**					
Hypertension (%)	48 (32.7%)	33 (51.6%)^a^	43 (43.0%)	56 (54.9%)^a^	0.003
Diabetes mellitus (%)	19 (12.9%)	6 (9.38%)	12 (12.0%)	18 (17.6%)	0.442
Atrial fibrillation (%)	2 (1.36%)	1 (1.56%)	2 (2.00%)	3 (2.94%)	0.895
FHS score	15 [12;18]	17 [15;19]^a^	16 [13;18]^b^	18 [16;20]^[Link]^, ^[Link]^	<0.001
*APOE* ɛ4 (%)	37 (25.2%)	14 (21.9%)	72 (72.0%)^[Link]^, ^[Link]^	60 (58.8%)^[Link]^, ^[Link]^	<0.001
**Medication usage**					
Anti‐dementia (%)	19 (12.9%)	14 (21.9%)	51 (51.0%)^[Link]^, ^[Link]^	47 (46.1%)^[Link]^, ^[Link]^	<0.001
Anti‐hypertension (%)	47 (32.0%)	27 (42.2%)	39 (39.0%)	50 (49.0%)	0.056

*Note*: Data were presented as mean (SD), *n* (%), or median (interquartile range).

Abbreviations: Aβ, amyloid beta; *APOE*, apolipoprotein E; FHS score, general Framingham Heart Study score; SD, standard deviation; WMH, white mattery hyperintensity.

^a^p, significantly different from Aβ−WMH−.

^b^p, significantly different from Aβ−WMH+.

^c^p, significantly different from Aβ+WMH−.

Compared to the control group, the Aβ+WMH+ group displayed lower Aβ42 levels, hippocampal volume, MMSE score, MoCA score, and higher p‐tau181 levels, pNfL levels, average SBP, PP, MAP, systolic BPV, PP variability, and MAP variability (*P* < 0.05; Table 2). In contrast, compared to the Aβ−WMH+ group, the Aβ+WMH+ group exhibited lower Aβ42 levels, MMSE score, MoCA score, and higher p‐tau181 levels and pNfL levels (*P* < 0.05). Last, compared to the Aβ+WMH− group, the Aβ+WMH+ group demonstrated higher WMH volume, pNfL levels, systolic BPV, PP variability, MAP variability, and lower MoCA score (*P* < 0.05).

**TABLE 2:** Blood pressure variability index, baseline biomarkers, and baseline cognition of different groups.

Indexes	Aβ−WMH− (*n* = 147)	Aβ−WMH+ (*n* = 64)	Aβ+WMH− (*n* = 100)	Aβ+WMH+ (*n* = 102)	*P*	*Adjusted P*
**BP and BPV index**						
Average SBP	129.7 (11.66)	136.0 (12.42)^a^	131.8 (13.56)	134.9 (13.69)^a^	0.001	0.332
SBP SD	7.74 (4.40)	10.0 (4.91)^a^	8.81 (4.28)	10.8 (5.86)^[Link]^, ^[Link]^	<0.001	0.001
SBP VIM	4.88 (2.76)	6.28 (3.05)^a^	5.54 (2.68)	6.79 (3.65)^[Link]^, ^[Link]^	<0.001	0.001
Average DBP	72.1 (7.21)	73.0 (7.22)	73.8 (7.85)	73.7 (7.28)	0.226	0.036
DBP SD	5.08 (2.68)	5.57 (2.63)	5.35 (2.17)	5.73 (2.79)	0.238	0.112
DBP VIM	4.93 (2.61)	5.41 (2.55)	5.20 (2.11)	5.56 (2.70)	0.239	0.112
Average PP	57.6 (9.8)	62.9 (10.99)^a^	58.0 (12.99)^b^	61.1 (12.91)^[Link]^, ^[Link]^	0.005	0.773
PP SD	7.21 (3.50)	8.90 (5.08)^a^	7.55 (3.94)^b^	9.00 (4.89)^[Link]^, ^[Link]^	0.002	0.066
PP VIM	4.85 (2.33)	5.92 (3.34)^a^	5.07 (2.61)	6.01 (3.21)^[Link]^, ^[Link]^	0.003	0.062
Average MAP	91.3 (7.65)	94.0 (7.7)	93.1 (8.06)	94.1 (7.8)^a^	0.018	0.069
MAP SD	5.17 (2.73)	6.00 (2.74)	5.66 (2.46)	6.72 (3.01)^[Link]^, ^[Link]^	<0.001	0.001
MAP VIM	3.77 (1.98)	4.37 (1.98)	4.12 (1.79)	4.89 (2.18)^[Link]^, ^[Link]^	<0.001	0.001
**Biomarkers**						
Aβ42	3.22 (0.13)	3.18 (0.13)^a^	2.84 (0.12)^[Link]^, ^[Link]^	2.81 (0.13)^[Link]^, ^[Link]^	<0.001	<0.001
Total tau	2.35 (0.15)	2.39 (0.15)	2.42 (0.21)^a^	2.45 (0.19)^a^	<0.001	<0.001
P‐tau181	1.29 (0.16)	1.34 (0.17)	1.41 (0.24)^[Link]^, ^[Link]^	1.44 (0.21)^[Link]^, ^[Link]^	<0.001	<0.001
Hippocampus	6.58 (0.83)	6.52 (0.93)	6.31 (0.84)^a^	6.26 (0.91)^a^	0.012	0.001
WMH	0.16 (0.35)	1.0 (0.27)^[Link]^, ^[Link]^	0.20 (0.32)	1.02 (0.28)^[Link]^, ^[Link]^	<0.001	<0.001
pNfL	1.44 (0.17)	1.54 (0.18)^a^	1.53 (0.18)^a^	1.62 (0.16)^[Link]^, ^[Link]^, ^[Link]^	<0.001	<0.001
**Cognition**						
MMSE	0.41 (0.59)	0.30 (0.59)	−0.03 (0.92)^[Link]^, ^[Link]^	−0.06 (0.86)^[Link]^, ^[Link]^	<0.001	<0.001
MoCA	0.32 (0.82)	0.17 (0.70)	−0.16 (0.84)^[Link]^, ^[Link]^	−0.41 (0.87)^[Link]^, ^[Link]^, ^[Link]^	<0.001	<0.001

*Notes*: WMH volume, pNfL, CSF Aβ42, total tau, and p‐tau181 levels were log transformed; MMSE and MoCA scores were *z* transformed; data were presented as mean (SD). Blood pressure was measured in mmHg, biomarkers were measured in pg/mL, and brain volume was measured in mL.

Abbreviations: Aβ, amyloid beta; BP, blood pressure; BPV, blood pressure variability; CSF, cerebrospinal fluid; DBP, diastolic blood pressure; MAP, mean arterial pressure; MMSE, Mini‐Mental State Examination; MoCA, Montreal Cognitive Assessment; pNfL, plasma neurofilament light; PP, pulse pressure; p‐tau, phosphorylated tau; SBP, systolic blood pressure; SD, standard deviation; VIM, variation independent of mean; WMH, white matter hyperintensities.

^a^p, significantly different from Aβ−WMH−.

^b^p, significantly different from Aβ−WMH+.

^c^p, significantly different from Aβ+WMH−.

### 3.2 Association of BPV or pNfL with brain structural MRI changes and cognition

In the total population, systolic BPV (VIM) exhibited associations with WMH volume (estimate [est] = 0.022, *P* = 0.004), memory function (est = −0.023, *P* = 0.042), and executive function (est = −0.030, *P* = 0.037; Table 3). Diastolic BPV (VIM) was linked to hippocampal volume (est = −0.053, *P* = 0.002), memory function (est = −0.029, *P* = 0.028), and executive function (est = −0.040, *P* = 0.008). PP variability (VIM) was associated with WMH volume (est = 0.023, *P* = 0.006) but not cognition. MAP variability (VIM) was associated with WMH volume (est = 0.027, *P* = 0.02), hippocampal volume (est = −0.057, *P* = 0.009), memory function (est = −0.037, *P* = 0.028), and executive function (est = −0.056, *P* = 0.004). Furthermore, pNfL demonstrated associations with WMH volume (est = 0.145, *P* < 0.001), hippocampal volume (est = −0.132, *P* = 0.014), MMSE score (est = −0.633, *P* < 0.001), MoCA score (est = −0.774, *P* < 0.001), memory function (est = −0.426, *P* < 0.001), and executive function (est = −0.662, *P* < 0.001).

**TABLE 3:** Longitudinal association of BPV with pNfL, brain structural changes, and cognition within 2 years.

Variables	SBP VIM	DBP VIM	PP VIM	MAP VIM	pNfL
Outcomes	est (*P*)	est (*P*)	est (*P*)	est (*P*)	est (*P*)
**Brain volume**					
WMH	0.022 (0.004)	0.154 (0.095)	0.023 (0.006)	0.027 (0.02)	0.145 (<0.001)
Hippocampus	−0.011 (0.424)	−0.053 (0.002)	0.007 (0.679)	−0.057 (0.009)	−0.132 (0.014)
**Cognition**					
MMSE	−0.020 (0.119)	−0.021 (0.152)	−0.016 (0.232)	−0.028 (0.127)	−0.633 (<0.001)
MoCA	−0.020 (0.127)	−0.026 (0.075)	−0.022 (0.103)	−0.028 (0.13)	−0.774 (<0.001)
Memory	−0.023 (0.042)	−0.029 (0.028)	−0.022 (0.073)	−0.037 (0.028)	−0.426 (<0.001)
Executive	−0.030 (0.037)	−0.040 (0.008)	−0.002 (0.863)	−0.056 (0.004)	−0.662 (<0.001)
**Biomarkers**					
pNfL	0.013 (<0.001)	0.012 (<0.001)	0.008 (0.009)	0.020 (<0.001)	/

Abbreviations: DBP, diastolic blood pressure; est, estimate; MAP, mean arterial pressure; MMSE, Mini‐Mental State Examination; MoCA, Montreal Cognitive Assessment; pNfL, plasma neurofilament light; PP, pulse pressure; SBP, systolic blood pressure; VIM, variation independent of mean; WMH, white matter hyperintensities.

### 3.3 Association of BPV with pNfL levels

**FIGURE 2:**
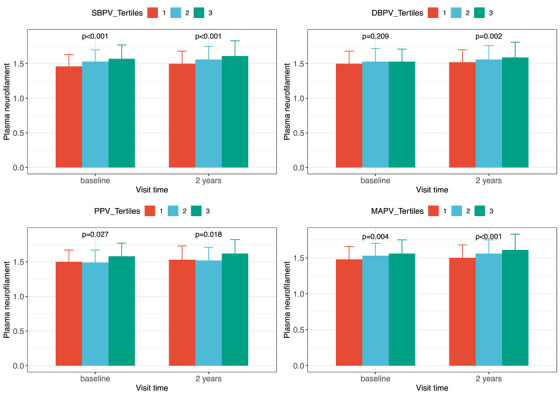
Plasma neurofilament light levels at baseline and 2 years among different blood pressure variability groups (by tertile). Note: BPV tertiles, level 1 represented the low tertile, level 2 represented the medium tertile, and level 3 represented the high tertile. DBPV, diastolic blood pressure; MAPV, mean arterial pressure variability; PPV, pulse pressure variability; SBPV, systolic blood pressure variability.

Baseline and 2‐year pNfL levels differed among individuals with varying systolic BPV (baseline and 2 years, all *P* < 0.001), PP variability (baseline *P* = 0.027 and 2‐year *P* = 0.018), and MAP variability (baseline *P* = 0.004 and 2‐year *P* < 0.001) tertiles (Figure 2) in the total population. There was no disparity in baseline pNfL levels among individuals with different diastolic BPV tertiles, but a significant difference in pNfL levels at 2 years (*P* = 0.002).

In linear mixed‐effects models, systolic BPV (est = 0.013, *P* < 0.001), diastolic BPV (est = 0.012, *P* < 0.001), PP variability (est = 0.008, *P* = 0.009), and MAP variability (est = 0.020, *P* < 0.001) were associated with pNfL levels (Table 3).

### 3.4 Association of BPV with brain structural MRI changes and cognition was mediated by pNfL

**FIGURE 3:**
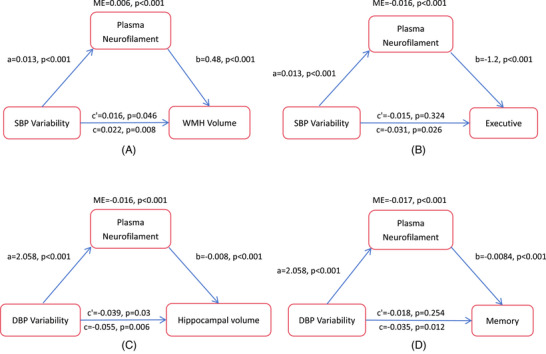
Association of blood pressure variability with brain structural changes and cognitive performance at 2 years was mediated by plasma neurofilament light. a, the effect of blood pressure variability on the mediator variable; b, the effect of the mediator variable on the outcome variable; c', direct effect; c, total effect; DBP, diastolic blood pressure; ME, mediation effect; SBP, systolic blood pressure; WMH, white matter hyperintensities.

In the total population, pNfL mediated the effect of systolic BPV on WMH volume and the effect of diastolic BPV on hippocampal volume (Figure 3). The impact of systolic BPV on executive function was mediated through pNfL, while the effect of diastolic BPV on memory function was similarly mediated by pNfL. No significant mediating effect of pNfL was observed on the association between systolic BPV and memory function or between diastolic BPV and executive function. Mediation analysis was not conducted due to the absence of significant associations between systolic BPV and hippocampal volume or between diastolic BPV and WMH volume. Moreover, pNfL mediated the effect of MAP variability on WMH/hippocampal volume and the effect of MAP variability on executive/memory function (Figure S2 in supporting information).

**FIGURE 4:**
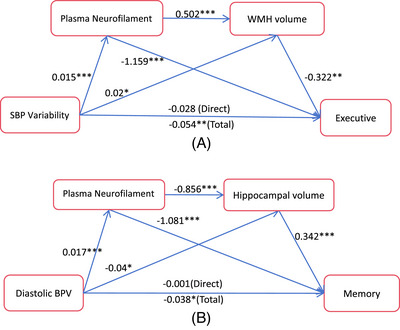
Association between blood pressure variability and cognition at 2 years was mediated by plasma neurofilament light and brain structural magnetic resonance imaging changes. Note: The association between systolic BPV and executive is potentially mediated by pNfL and WMH volume through three indirect effects (4A), path 1: systolic BPV → pNfL → executive (−0.017), path 2: systolic BPV → WMH volume → executive (−0.006), and path 3: systolic BPV → pNfL → WMH volume → executive (−0.003). Path 1 and path 3 demonstrated statistical significance, while path 2 did not reach a significant level (*P* = 0.071). Similarly, the mediating effect of pNfL and hippocampal volume on the association between diastolic BPV and memory can be attributed to three indirect effects (4B), path 1: diastolic BPV → pNfL → memory (−0.018), path 2: diastolic BPV → hippocampal volume → memory (−0.014), and path 3: diastolic BPV → pNfL → hippocampal volume → memory (−0.005). Path 1 and path 3 exhibited a significant level of association, whereas path 2 did not reach statistical significance (*P* = 0.061). ^*^ *P* < 0.05, ^**^ *P* < 0.01, ^***^ *P* < 0.001. BPV, blood pressure variability; pNfL, plasma neurofilament light; WMH, white matter hyperintensities.

Chain mediation analyses revealed that the association between systolic BPV and executive function was mediated by pNfL and WMH volume, while the association between diastolic BPV and memory function was mediated by pNfL and hippocampal volume (Figure 4). Likewise, the association between MAP variability and executive function was mediated by pNfL and WMH volume, and the association between MAP variability and memory function was mediated by pNfL and hippocampal volume (Figure S3 in supporting information).

### 3.5 Supplementary analyses

When we defined the presence of either lacunar infarcts or a high WMH burden as indicative of CSVD pathology, the differences in biomarkers among different disease groups were consistent with the previous findings (Table S1 in supporting information).

Logistic regression analysis was used to assess the relationship between BPV and the risk of comorbidity between AD and CSVD at 2 years. The results indicated that systolic BPV (odds ratio [OR] 1.24, 95% confidence interval [CI] 1.09 to 1.42, *P* = 0.002) and MAP variability (OR 1.39, 95% CI 1.15 to 1.71) were associated with higher odds of comorbidity between AD and CSVD (Table S2 in supporting information).

When these statistical analyses (comparing the difference of pNfL among different BPV tertiles, linear mixed‐effects models, and mediation analysis) were conducted in different disease groups or Aβ status, the results demonstrated that the association of BPV with brain structure and cognitive function and the mediating effect of pNfL in the relationship between BPV and brain structural changes and cognitive function was not as significant as that in the total population (Tables S3–S8, Figures S4–S15 in supporting information).

## 4 DISCUSSION

In this study, we examined the association between long‐term BPV, pNfL, and the comorbidity of AD and CSVD in individuals with normal cognition and MCI. Our findings revealed the following key points: (1) Aβ‐positive individuals with a high WMH burden, which signifies the comorbidity of AD and CSVD, displayed elevated BPV and pNfL levels; (2) in the total population, BPV was associated with pNfL levels, brain structural changes related to comorbidity (including hippocampal atrophy and WMH progression), cognitive performance, and the risk of comorbidity independently of average BP; (3) pNfL played a mediating role in the relationship between BPV, comorbidity‐related brain structural changes, and cognition. These findings emphasize the potential effects of BPV and pNfL on the mechanisms of comorbidity between AD and CSVD.

Previous studies have shown an association between elevated BPV and pNfL with Aβ pathology^11^, ^30^ and WMH progression.^7^, ^31^ In the present study, we observed that Aβ‐positive individuals with a high WMH burden exhibited higher BPV and pNfL levels than other groups. Therefore, the speculation that BPV and pNfL are associated with the comorbidity of AD and CSVD has preliminarily been confirmed. We further investigated the association of BPV or pNfL with imaging markers of comorbidity (hippocampal and WMH volumes) and cognitive performance. We found that elevated systolic BPV was associated with higher WMH volume, while elevated diastolic BPV was linked to lower hippocampal volume. Our study findings align with some previous reports,^10^, ^32^, ^33^, ^34^ but other studies have shown no association between systolic BPV and WMH volume,^35^ nor between diastolic BPV and hippocampal volume.^36^ A possible explanation for our finding is that SBP and DBP have differential effects on different regions of the brain. White matter is mainly supplied by perforating arteries perpendicular to the brain surface, and larger variability in SBP is more likely to cause pathological changes in perforating arteries,^37^ thus having a greater impact on white matter lesions. However, the hippocampus is supplied with blood by the anterior choroidal artery and posterior cerebral artery,^38^ which are closely associated with cerebral blood flow.^39^ Previous studies suggest that the impact of DBP on cerebral perfusion was greater than that of SBP.^40^ Therefore, large fluctuations in DBP may have a greater impact on the hippocampus. MAP variability combined the effects of both systolic and diastolic BPV, and was related to both WMH and hippocampal volumes. The relationship between BPV and cognition,^33^, ^34^ as well as the association of pNfL with imaging markers and cognition,^20^, ^21^ were consistent with prior studies. Through supplementary analyses, we also found that elevated BPV was associated with an increased risk of comorbidity at 2 years. In summary, our study further strengthens the association between BPV, pNfL, and the comorbidity of AD and CSVD.

The underlying mechanism by which BPV's role is implicated in the pathogenesis of the comorbidity between AD and CSVD is poorly understood. Evidence suggests that BPV may be associated with axonal injury,^10^, ^13^ and pNfL serves as a reliable indicator reflecting axonal injury.^16^ In this study, we evaluated the relationship between BPV and pNfL levels and found that individuals with higher BPV had higher pNfL levels. This result further supports the concept of BPV being associated with axonal injury. Evidence indicates that elevated BPV acts as a risk factor for AD,^6^ with axonal injury initiating as an early event in AD,^14^ and changes in brain structure and cognition manifest in later stages.^15^ Building upon the chronological sequence of these events, we performed causal mediation analyses and found that pNfL mediated the effects of BPV on brain structural changes and cognition in the total population. Additionally, chain mediation analyses revealed that the association between BPV and cognition at 2 years was mediated by pNfL and brain structural changes. Previous studies have indicated that elevated BPV is linked to various health issues, including arterial sclerosis, injury to the brain's microvasculature,^10^ reduced cerebral perfusion,^41^ inflammation activation, and Aβ pathology,^11^ among others.^42^ These mechanisms, notably chronic hypoxia and Aβ pathology, may result in axonal injury.^43^, ^44^ In simpler terms, it is possible that elevated BPV could potentially contribute to axonal injury through the above mechanisms. Overall, the above evidence may suggest that BPV could potentially cause macro‐level changes in brain structure and cognitive performance by inducing micro‐level axonal injury. However, across different disease groups, the mediating effect of pNfL was not significant, which may be attributed to the small sample sizes in each group (ranging from 64 to 147 cases).^45^ Furthermore, when conducting mediation analysis only within the comorbidity group, the mediating effect of pNfL may be confounded by pre‐existing significant Aβ and vascular pathology at baseline.^44^, ^46^ Thus, our results may be more applicable to the general population. Future studies with larger sample sizes, particularly focusing on comorbidity, can further investigate the mediating effect of NFL.

Importantly, pNfL played a role in mediating the relationship between different components of BPV and changes in brain structure and cognition. To clarify, pNfL mediated the effects of systolic BPV on WMH volume and executive function, the effects of diastolic BPV on hippocampal volume and memory function, and the effects of MAP variability on WMH/hippocampal volume and executive/memory function. These results suggest that distinct components of BPV might lead to specific regional changes in brain structure and cognitive decline. Additionally, we observed that systolic BPV and diastolic BPV showed good consistency (Table S9 in supporting information). These findings imply that elevated BPV, as a common risk factor, may be associated with pathological changes in brain regions related to AD and CSVD, and thereby may contribute to the comorbidity of AD and CSVD. Furthermore, as BPV increases, the presence of pure AD or CSVD pathology may further accelerate axonal injury,^14^, ^47^ resulting in more severe hippocampal atrophy or white matter lesions. Altogether, our study identifies a potential association between BPV and axonal injury, enhancing our understanding of the underlying pathological mechanisms of BPV and the mechanisms behind the comorbidity of AD and CSVD.

Several limitations should be acknowledged in our study. First, we focused on WMH as the representative marker of CSVD, which may not fully capture the complexity of CSVD. However, our supplementary analysis consistently yielded similar results, irrespective of whether we used WMH alone or in combination with lacunar infarcts to define CSVD pathology. Second, the data used in this study were retrospectively collected from the ADNI database, which may introduce some biases, and the sample size was insufficient. We have only confirmed our hypothesis in the total population and have not confirmed this hypothesis across different disease groups or Aβ status. Future longitudinal studies need to validate this hypothesis in comorbidity. Last, because our mediation analyses were based on observed causal patterns in epidemiology, caution is needed when interpreting causal associations. Long‐term BPV's association with pNfL would require longer follow‐up studies. Nevertheless, our study has notable strengths, offering new insights into the relationship between long‐term BPV and micro‐ and macro‐level changes in the comorbidity of AD and CSVD. This discovery has implications for the prevention and treatment of AD, underscoring the importance of early BPV management.

In conclusion, our findings suggest a connection between BPV and pNfL levels. In the general population, this connection may be associated with hippocampal atrophy, WMH progression, and cognitive decline, which are hallmarks of the comorbidity of AD and CSVD. Our study offers valuable evidence for comprehending how a common risk factor, like elevated BPV, may contribute to pathological changes in diverse brain regions associated with both AD and CSVD and cognitive decline, potentially linking to the comorbidity. In the future, research efforts should prioritize BPV management to enhance dementia prevention, especially in AD patients displaying typical vascular pathology.

## CONFLICT OF INTEREST STATEMENT

The authors declare no conflicts of interest. Author disclosures are available in the supporting information.

## CONSENT STATEMENT

The ADNI study was approved by the institutional review boards of all participating institutions. Written informed consent was obtained from all the participants or their authorized representatives following the Declaration of Helsinki.

## Supporting information

Supporting Information

Supporting Information
