# Supplementary material for: Potential role of blood pressure variability and plasma neurofilament light in the mechanism of comorbidity between Alzheimer's disease and cerebral small vessel disease
Source: Alzheimers Dement. 2024 Jun 19;20(7):4891–902. doi: 10.1002/alz.14056 (PMC11247680; doi:10.1002/alz.14056)
Supplement: Supplementary file 1 — Supporting Information [file ALZ-20-4891-s001.docx]

**Potential Role of Blood Pressure Variability and Plasma Neurofilament Light in the Mechanism of Comorbidity Between Alzheimer’s Disease and Cerebral Small Vessel Disease**

**SUPPLEMENTAL MATERIAL**

Table S1 Supplement analyses of accessory examination results and blood pressure variability index of different groups

| Indexes | Aβ-CSVD-  (n = 138) | Aβ-CSVD+  (n = 73) | Aβ+CSVD-  (n = 97) | Aβ+CSVD+  (n = 105) | *p* | *p adjusted* |
| --- | --- | --- | --- | --- | --- | --- |
| **Accessory examination** | | | | | | |
| Aβ42 | 3.22 (0.13) | 3.18 (0.14) | 2.84 (0.12) *† | 2.81 (0.13) *† | <0.001 | <0.001 |
| Total tau | 2.35 (0.14) | 2.38 (0.16) | 2.42 (0.21) * | 2.45 (0.19) * | <0.001 | <0.001 |
| P-tau181 | 1.29 (0.16) | 1.33 (0.18) | 1.40 (0.23) * | 1.44 (0.21) *† | <0.001 | <0.001 |
| Hippocampus | 6.57 (0.85) | 6.55 (0.88) | 6.30 (0.85) | 6.26 (0.90) * | 0.013 | 0.002 |
| WMH | 0.15 (0.35) | 0.91 (0.38) * | 0.19 (0.33) † | 1.00 (0.30) *‡ | <0.001 | <0.001 |
| PNFL | 1.44 (0.16) | 1.53 (0.20) * | 1.53 (0.18) * | 1.62 (0.16) *†‡ | <0.001 | <0.001 |
| **BP and BPV index** | | | | | | |
| Average SBP | 129 (11.5) | 136 (12.4) * | 132 (13.7) | 135 (13.6) * | 0.001 | 0.306 |
| SBP SD | 7.74 (4.49) | 9.74 (4.74) * | 8.88 (4.27) | 10.7 (5.86) *‡ | <0.001 | 0.002 |
| SBP VIM | 4.88 (2.82) | 6.10 (2.94) * | 5.58 (2.67) | 6.71 (3.65) * | <0.001 | 0.002 |
| Average DBP | 71.9 (7.13) | 73.3 (7.32) | 73.9 (7.88) | 73.6 (7.27) | 0.134 | 0.034 |
| DBP SD | 5.06 (2.73) | 5.55 (2.54) | 5.36 (2.15) | 5.71 (2.79) | 0.241 | 0.106 |
| DBP VIM | 4.91 (2.65) | 5.38 (2.47) | 5.20 (2.08) | 5.55 (2.71) | 0.242 | 0.107 |
| Average PP | 57.4 (9.78) | 62.5 (10.9) | 58.0 (13.1) | 61.0 (12.8) | 0.006 | 0.81 |
| PP SD | 7.29 (3.48) | 8.55 (5.00) | 7.66 (3.94) | 8.87 (4.90) | 0.02 | 0.133 |
| PP VIM | 4.90 (2.32) | 5.69 (3.28) | 5.14 (2.61) | 5.92 (3.22) | 0.025 | 0.108 |
| Average MAP | 91.0 (7.53) | 94.2 (7.80) * | 93.3 (8.09) | 93.9 (7.79) * | 0.008 | 0.063 |
| MAP SD | 5.14 (2.79) | 5.95 (2.61) | 5.67 (2.44) | 6.68 (3.02) * | <0.001 | 0.001 |
| MAP VIM | 3.75 (2.03) | 4.33 (1.89) | 4.13 (1.78) | 4.86 (2.19) * | <0.001 | 0.001 |
| **Cognition** | | | | | | |
| MMSE | 0.42 (0.59) | 0.31 (0.58) | -0.04 (0.93) *† | -0.05 (0.85) *† | <0.001 | <0.001 |
| MoCA | 0.29 (0.82) | 0.24 (0.73) | -0.17 (0.84) * | -0.40 (0.87) *† | <0.001 | <0.001 |

Note: WMH volume, PNFL, CSF Aβ42, total tau, and p-tau181 levels were log-transformed; MMSE and MoCA scores were z-transformed; data were presented as mean (SD). Blood pressure was measured in mmHg, biomarkers were measured in pg/ml, and brain volume was measured in ml. Abbreviation: CSVD, cerebral small vessel disease; WMH, white matter hyperintensities; PNFL, plasma neurofilament light; SBP, systolic blood pressure; DBP, diastolic blood pressure; PP, pulse pressure; MAP, mean arterial pressure; SD, standard deviation; VIM, variation independent of mean; MMSE, Mini-Mental State Examination; MoCA, Montreal Cognitive Assessment; * significant represented the difference between Aβ-CSVD- and other groups; † significant represented the difference between Aβ-CSVD+ and Aβ+CSVD- or Aβ+CSVD+; ‡ significant represented the difference between Aβ+CSVD- and Aβ+CSVD+.

Table S2 Relationship between blood pressure variability and the risk of the comorbidity of Alzheimer’s disease and cerebral small vessel disease

| Outcomes | Comorbidity  (Aβ+WMH+, N = 249) | | | Comorbidity  (Atrophy+WMH+, N = 413) | | |
| --- | --- | --- | --- | --- | --- | --- |
| Predictors | OR | 95%CI | *p* | OR | 95%CI | *p* |
| SBP VIM | 1.24 | (1.09, 1.42) | 0.002 | 1.11 | (1.02, 1.21) | 0.012 |
| DBP VIM | 1.13 | (0.97, 1.31) | 0.112 | 1.17 | (1.06, 1.29) | 0.001 |
| PP VIM | 1.11 | (0.99, 1.25) | 0.068 | 1.07 | (0.98, 1.17) | 0.116 |
| MAP VIM | 1.39 | (1.15, 1.71) | 0.001 | 1.24 | (1.10, 1.40) | <0.001 |

Abbreviation: OR, odds ratio; CI, confidence interval; SBP, systolic blood pressure; DBP, diastolic blood pressure; MAP, mean arterial pressure; VIM, variation independent of mean. Comorbidity was determined through Aβ-positive with high WMH burden or imaging biomarkers (high white matter hypertensities burden and hippocampal atrophy). Hippocampal atrophy was defined based on median (hippocampal volume < median volume). Logistic regression was adjusted for FHS score, APOE ε4 status, cognitive status, Aβ status, the use of anti-hypertension medications, and average BP.

Table S3 Longitudinal association of BPV with PNFL, brain structural changes, and cognition within 2 years in the Aβ-WMH- group

| Variables | SBP VIM | DBP VIM | PP VIM | MAP VIM | PNFL |
| --- | --- | --- | --- | --- | --- |
| Outcomes | est (*p*) | est (*p*) | est (*p*) | est (*p*) | est (*p*) |
| **Brain volume** |  |  |  |  |  |
| WMH | -0.005  (0.663) | 0.010  (0.379) | -0.004  (0.767) | -0.017  (0.256) | 0.148  (0.015) |
| Hippocampus | -0.023  (0.387) | -0.047  (0.075) | -0.013  (0.681) | -0.058  (0.100) | -0.053  (0.511) |
| **Cognition** |  |  |  |  |  |
| MMSE | -0.011  (0.432) | -0.016  (0.301) | -0.022  (0.210) | -0.017  (0.382) | -0.408  (0.023) |
| MoCA | -0.001  (0.962) | -0.025  (0.226) | -0.024  (0.304) | -0.009  (0.727) | -0.772  (<0.001) |
| Memory | -0.015  (0.446) | -0.010  (0.619) | -0.044  (0.060) | -0.007  (0.790) | -0.418  (0.007) |
| Executive | -0.015  (0.494) | -0.044  (0.047) | 0.014  (0.584) | -0.053  (0.073) | -0.819  (<0.001) |
| **Biomarkers** |  |  |  |  |  |
| PNFL | 0.017  (0.001) | 0.012  (0.030) | 0.014  (0.031) | 0.022  (0.002) | / |

Abbreviation: est, estimate; SBP, systolic blood pressure; DBP, diastolic blood pressure; PP, pulse pressure; MAP, mean arterial pressure; VIM, variation independent of mean; WMH, white matter hyperintensities; MMSE, Mini-Mental Statement Examination; MoCA, Montreal Cognitive Assessment; PNFL, plasma neurofilament light.

Table S4 Longitudinal association of BPV with PNFL, brain structural changes, and cognition within 2 years in the Aβ-WMH+ group

| Variables | SBP VIM | DBP VIM | PP VIM | MAP VIM | PNFL |
| --- | --- | --- | --- | --- | --- |
| Outcomes | est (*p*) | est (*p*) | est (*p*) | est (*p*) | est (*p*) |
| **Brain volume** |  |  |  |  |  |
| WMH | 0.010  (0.384) | 0.012  (0.379) | 0.007  (0.502) | 0.021  (0.213) | 0.029  (0.741) |
| Hippocampus | 0.032  (0.448) | -0.050  (0.297) | 0.081  (0.027) | -0.055  (0.351) | -0.110  (0.436) |
| **Cognition** |  |  |  |  |  |
| MMSE | -0.016  (0.602) | -0.035  (0.309) | 0.030  (0.257) | -0.076  (0.069) | -0.429  (0.246) |
| MoCA | -0.013  (0.629) | -0.037  (0.220) | 0.003  (0.898) | -0.041  (0.275) | -0.417  (0.181) |
| Memory | -0.010  (0.739) | -0.009  (0.778) | 0.002  (0.941) | -0.022  (0.596) | -0.471  (0.060) |
| Executive | 0.006  (0.845) | -0.003  (0.919) | 0.009  (0.734) | -0.008  (0.839) | -0.597  (0.048) |
| **Biomarkers** |  |  |  |  |  |
| PNFL | 0.0004  (0.965) | 0.013  (0.199) | -0.004  (0.644) | 0.018  (0.160) | / |

Abbreviation: est, estimate; SBP, systolic blood pressure; DBP, diastolic blood pressure; PP, pulse pressure; MAP, mean arterial pressure; VIM, variation independent of mean; WMH, white matter hyperintensities; MMSE, Mini-Mental Statement Examination; MoCA, Montreal Cognitive Assessment; PNFL, plasma neurofilament light.

Table S5 Longitudinal association of BPV with PNFL, brain structural changes, and cognition within 2 years in the Aβ+WMH- group

| Variables | SBP VIM | DBP VIM | PP VIM | MAP VIM | PNFL |
| --- | --- | --- | --- | --- | --- |
| Outcomes | est (*p*) | est (*p*) | est (*p*) | est (*p*) | est (*p*) |
| **Brain volume** |  |  |  |  |  |
| WMH | 0.001  (0.928) | 0.018  (0.221) | 0.011  (0.354) | 0.007  (0.682) | 0.298  (<0.001) |
| Hippocampus | 0.003  (0.937) | -0.063  (0.120) | -0.008  (0.817) | -0.035  (0.469) | -0.343  (0.009) |
| **Cognition** |  |  |  |  |  |
| MMSE | 0.008  (0.826) | 0.053  (0.246) | -0.011  (0.769) | 0.057  (0.282) | -0.875  (0.037) |
| MoCA | -0.035  (0.320) | 0.021  (0.630) | -0.035  (0.332) | -0.002  (0.972) | -1.26  (<0.001) |
| Memory | -0.036  (0.257) | -0.004  (0.918) | -0.039  (0.225) | -0.028  (0.537) | -0.456  (0.065) |
| Executive | -0.077  (0.037) | -0.010  (0.823) | -0.032  (0.409) | -0.067  (0.210) | -0.620  (0.072) |
| **Biomarkers** |  |  |  |  |  |
| PNFL | 0.012  (0.072) | 0.005  (0.558) | 0.007  (0.301) | 0.008  (0.386) | / |

Abbreviation: est, estimate; SBP, systolic blood pressure; DBP, diastolic blood pressure; PP, pulse pressure; MAP, mean arterial pressure; VIM, variation independent of mean; WMH, white matter hyperintensities; MMSE, Mini-Mental Statement Examination; MoCA, Montreal Cognitive Assessment; PNFL, plasma neurofilament light.

Table S6 Longitudinal association of BPV with PNFL, brain structural changes, and cognition within 2 years in the Aβ+WMH+ group

| Variables | SBP VIM | DBP VIM | PP VIM | MAP VIM | PNFL |
| --- | --- | --- | --- | --- | --- |
| Outcomes | est (*p*) | est (*p*) | est (*p*) | est (*p*) | est (*p*) |
| **Brain volume** |  |  |  |  |  |
| WMH | 0.0001 (0.986) | 0.006  (0.600) | -0.002 (0.864) | 0.005  (0.688) | 0.054  (0.338) |
| Hippocampus | -0.014  (0.598) | -0.007  (0.829) | -0.006 (0.840) | -0.027 (0.535) | -0.136 (0.160) |
| **Cognition** |  |  |  |  |  |
| MMSE | -0.031  (0.242) | -0.020  (0.056) | -0.033 (0.259) | -0.035 (0.407) | -0.790 (0.038) |
| MoCA | -0.019  (0.423) | -0.015  (0.621) | -0.017 (0.503) | -0.025 (0.513) | -0.450 (0.177) |
| Memory | -0.014  (0.440) | -0.004  (0.109) | -0.006 (0.936) | -0.043 (0.139) | -0.338 (0.056) |
| Executive | 0.0003 (0.989) | -0.039  (0.196) | 0.021  (0.404) | -0.042 (0.248) | -0.379 (0.150) |
| **Biomarkers** |  |  |  |  |  |
| PNFL | 0.007  (0.143) | 0.007 (00.218) | 0.007  (0.183) | 0.013  (0.079) | / |

Abbreviation: est, estimate; SBP, systolic blood pressure; DBP, diastolic blood pressure; PP, pulse pressure; MAP, mean arterial pressure; VIM, variation independent of mean; WMH, white matter hyperintensities; MMSE, Mini-Mental Statement Examination; MoCA, Montreal Cognitive Assessment; PNFL, plasma neurofilament light.

Table S7 Longitudinal association of BPV with PNFL, brain structural changes, and cognition within 2 years in Aβ-positive individuals

| Variables | SBP VIM | DBP VIM | PP VIM | MAP VIM | PNFL |
| --- | --- | --- | --- | --- | --- |
| Outcomes | est (*p*) | est (*p*) | est (*p*) | est (*p*) | est (*p*) |
| **Brain volume** |  |  |  |  |  |
| WMH | 0.021  (0.038) | 0.028  (0.042) | 0.020  (0.07) | -0.044  (0.006) | 0.156  (0.005) |
| Hippocampus | -0.011  (0.565) | -0.032  (0.210) | -0.008  (0.718) | -0.037  (0.227) | -0.226  (0.004) |
| **Cognition** |  |  |  |  |  |
| MMSE | -0.019  (0.367) | 0.003  (0.902) | -0.026  (0.253) | -0.003  (0.927) | -0.842  (0.002) |
| MoCA | -0.029  (0.130) | -0.010  (0.698) | -0.028  (0.178) | -0.025  (0.397) | -0.844  (<0.001) |
| Memory | -0.027  (0.094) | -0.025  (0.226) | -0.020  (0.243) | -0.041  (0.101) | -0.384  (0.009) |
| Executive | -0.031  (0.118) | -0.035  (0.161) | -0.003  (0.876) | -0.060  (0.048) | -0.515  (0.014) |
| **Biomarkers** |  |  |  |  |  |
| PNFL | 0.011  (0.004) | 0.009  (0.072) | 0.009  (0.032) | 0.015  (0.01) | / |

Abbreviation: est, estimate; SBP, systolic blood pressure; DBP, diastolic blood pressure; PP, pulse pressure; MAP, mean arterial pressure; VIM, variation independent of mean; WMH, white matter hyperintensities; MMSE, Mini-Mental Statement Examination; MoCA, Montreal Cognitive Assessment; PNFL, plasma neurofilament light.

Table S8 Longitudinal association of BPV with PNFL, brain structural changes, and cognition within 2 years in Aβ-negative individuals

| Variables | SBP VIM | DBP VIM | PP VIM | MAP VIM | PNFL |
| --- | --- | --- | --- | --- | --- |
| Outcomes | est (*p*) | est (*p*) | est (*p*) | est (*p*) | est (*p*) |
| **Brain volume** |  |  |  |  |  |
| WMH | 0.023  (0.062) | 0.007  (0.579) | 0.023  (0.076) | 0.013  (0.46) | 0.146  (0.006) |
| Hippocampus | -0.009  (0.693) | -0.055  (0.016) | 0.026  (0.264) | -0.063  (0.04) | -0.066  (0.351) |
| **Cognition** |  |  |  |  |  |
| MMSE | -0.013  (0.321) | -0.027  (0.053) | -0.001  (0.926) | -0.037  (0.044) | -0.428  (0.01) |
| MoCA | -0.007  (0.659) | -0.029  (0.07) | -0.015  (0.355) | -0.021  (0.337) | -0.682  (<0.001) |
| Memory | -0.015  (0.336) | -0.017  (0.32) | -0.027  (0.106) | -0.16  (0.464) | -0.455  (<0.001) |
| Executive | -0.022  (0.219) | -0.043  (0.02) | 0.001  (0.968) | -0.051  (0.036) | -0.812  (<0.001) |
| **Biomarkers** |  |  |  |  |  |
| PNFL | 0.014  (0.002) | 0.014  (0.005) | 0.007  (0.130) | 0.023  (<0.001) | / |

Abbreviation: est, estimate; SBP, systolic blood pressure; DBP, diastolic blood pressure; PP, pulse pressure; MAP, mean arterial pressure; VIM, variation independent of mean; WMH, white matter hyperintensities; MMSE, Mini-Mental Statement Examination; MoCA, Montreal Cognitive Assessment; PNFL, plasma neurofilament light.

Table S9 Diastolic or systolic BPV of different systolic or diastolic BPV tertile groups

| Group | Low tertile | Medium tertile | High tertile | *p* |
| --- | --- | --- | --- | --- |
| SBP tertiles |  |  |  |  |
| DBP VIM | 4.70 (2.42) | 4.86 (2.26) | 6.11 (2.63) *† | <0.001 |
| DBP tertiles |  |  |  |  |
| SBP VIM | 4.99 (2.38) | 5.41 (2.67) | 6.78 (3.83) *† | <0.001 |

Note: *, significantly different from low tertile; †, significantly different from medium tertile. Abbreviation: SBP, systolic blood pressure; DBP, diastolic blood pressure; VIM, variation independent of mean; BPV, blood pressure variability.

Figure S1 Flowchart of participant data selection process


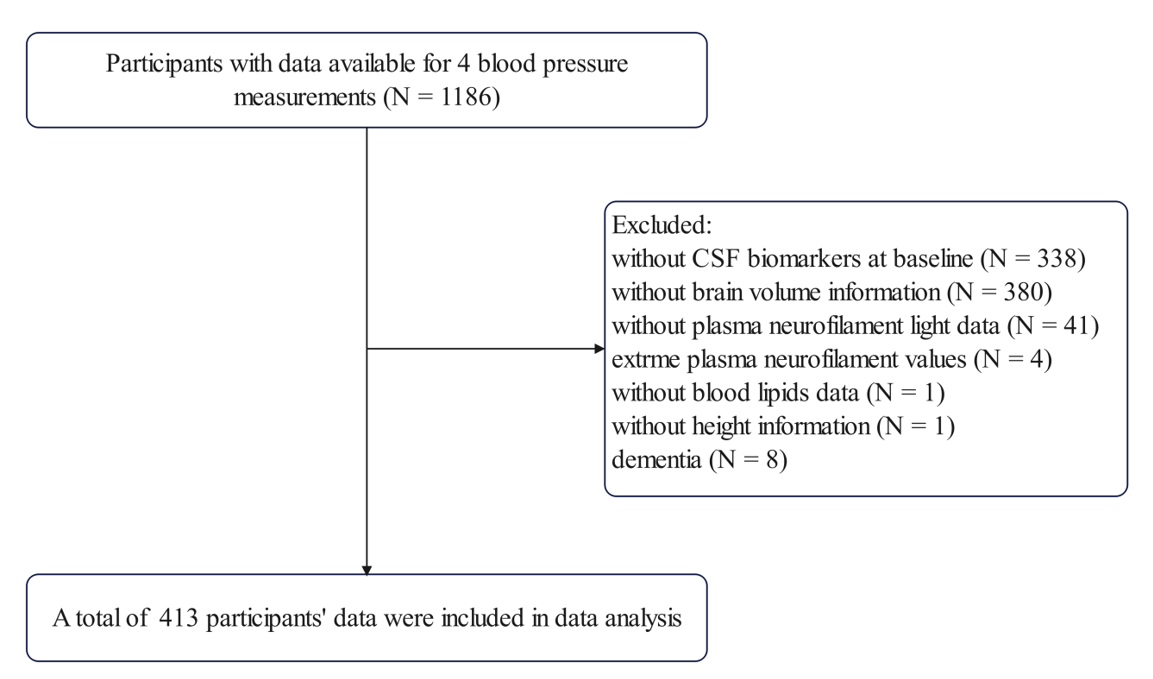


Abbreviation: CN, cognitively normal; MCI, mild cognitive impairment.

Figure S2 Association of mean arterial pressure variability with brain structural changes and cognitive performance at 2 years was mediated by plasma neurofilament light


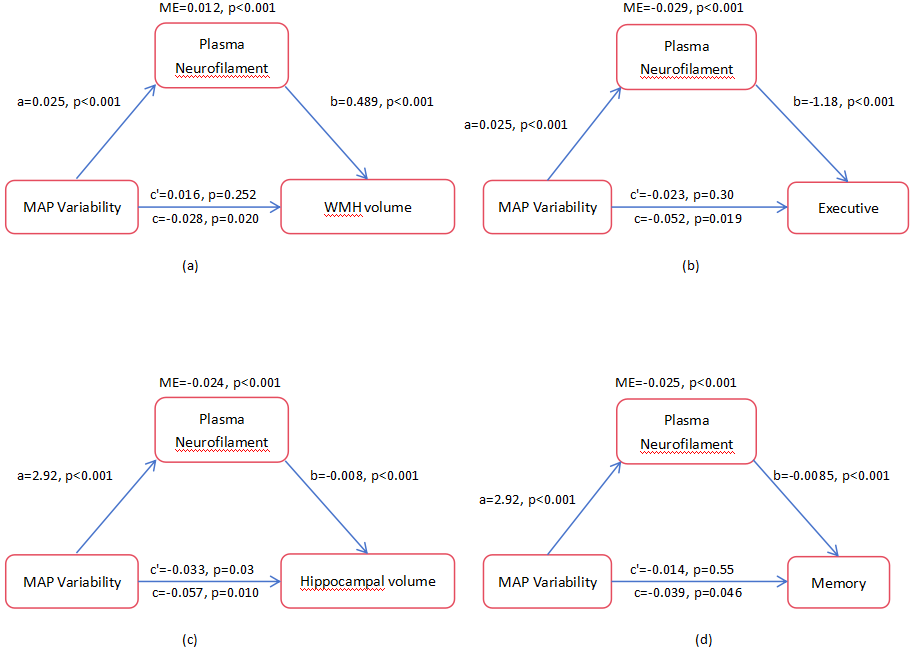
Note: ME, mediation effect; a, the effect of blood pressure variability on the mediator variable; b, the effect of the mediator variable on the outcome variable; c', direct effect; c, total effect; MAP, mean arterial pressure.

Figure S3 Association between mean arterial pressure variability and cognition at 2 years was mediated by plasma neurofilament light and brain structural MRI changes


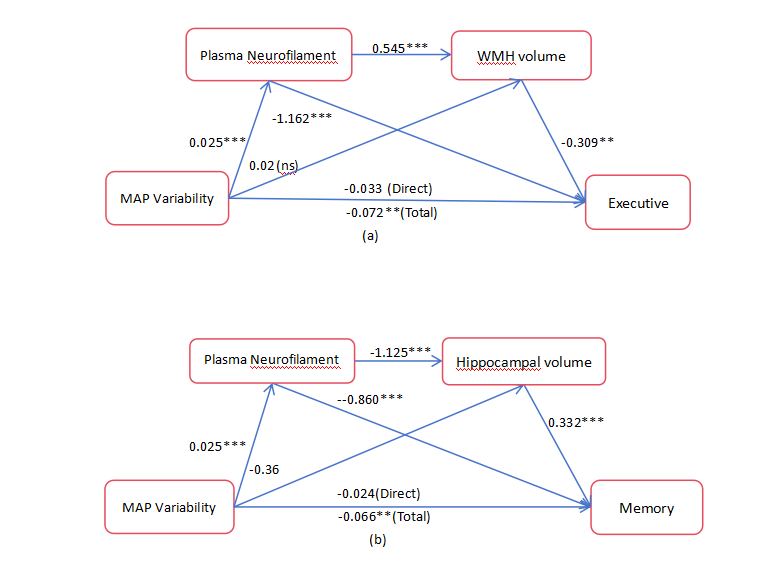


Note: MAP, mean arterial pressure; WMH, white matter hyperintensities. (a) The association between MAP variability and executive was mediated by PNFL and WMH volume through three indirect effects, path 1: MAP variability → PNFL → executive (-0.029), path 2: MAP variability → WMH volume → executive (-0.006), and path 3: MAP variability → PNFL → WMH volume → executive (-0.004, p=0.032). Path 1 and path 3 demonstrated statistical significance, while path 2 and direct effect did not reach a significant level. Abbreviation: WMH, white matter hyperintensities. (b) The association between MAP variability and memory was mediated by PNFL and hippocampal volume through three indirect effects path 1: MAP variability → PNFL → memory (-0.021), path 2: MAP variability → hippocampal volume → memory (-0.012), and path 3: MAP variability → PNFL → hippocampal volume → memory (-0.009). Path 1 and path 3 exhibited a significant level of association, whereas path 2 and direct effect did not reach statistical significance. *p < 0.05, **p < 0.01, ***p < 0.001.

Figure S4 Plasma neurofilament light Levels at baseline and 2 years among different blood pressure variability groups (by Tertile) in Aβ-WMH- group


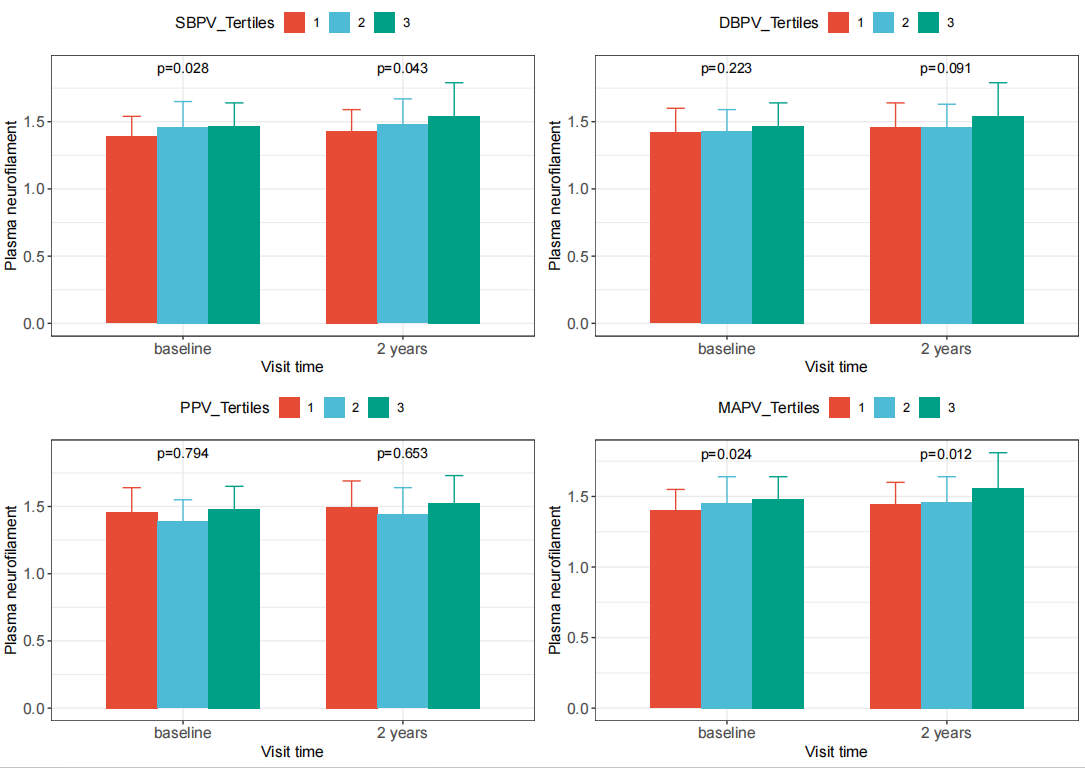


Note: BPV tertiles, level 1 represented the low tertile, level 2 represented the medium tertile, and level 3 represented the high tertile. SBPV, systolic blood pressure variability; DBPV, diastolic blood pressure; PPV, pulse pressure variability; MAPV, mean arterial pressure variability.

Figure S5 Plasma neurofilament light Levels at baseline and 2 years among different blood pressure variability groups (by Tertile) in Aβ-WMH+ group


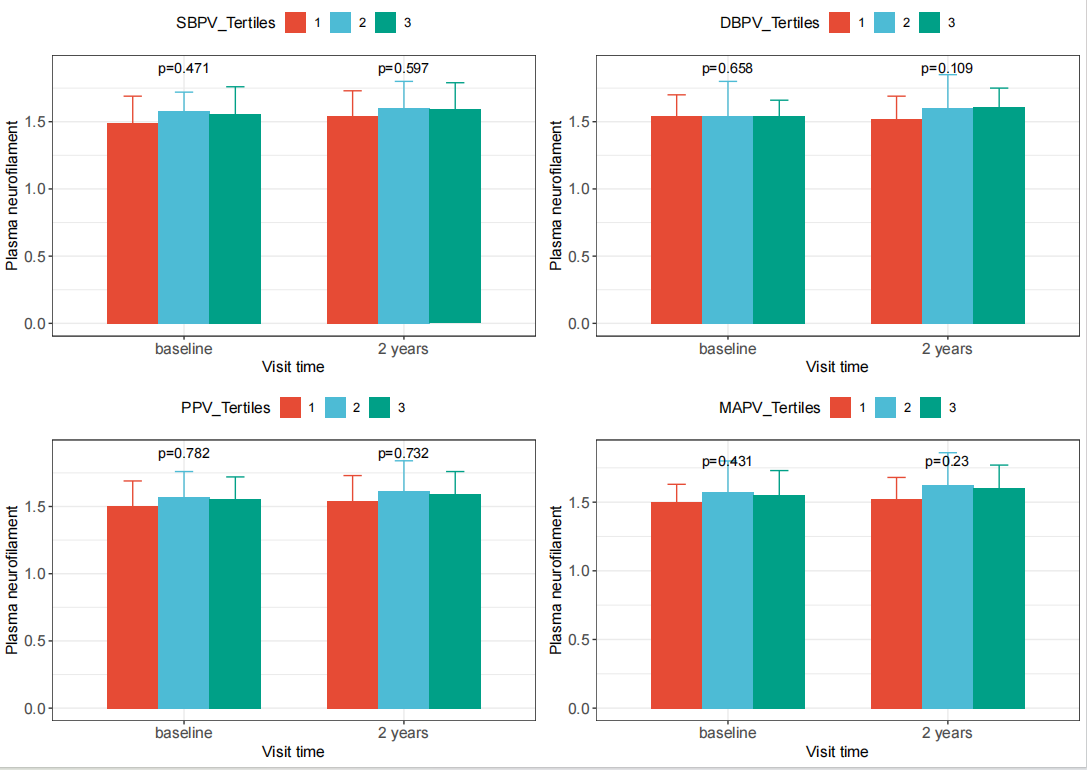


Note: BPV tertiles, level 1 represented the low tertile, level 2 represented the medium tertile, and level 3 represented the high tertile. SBPV, systolic blood pressure variability; DBPV, diastolic blood pressure; PPV, pulse pressure variability; MAPV, mean arterial pressure variability.

Figure S6 Plasma neurofilament light Levels at baseline and 2 years among different blood pressure variability groups (by Tertile) in Aβ+WMH- group


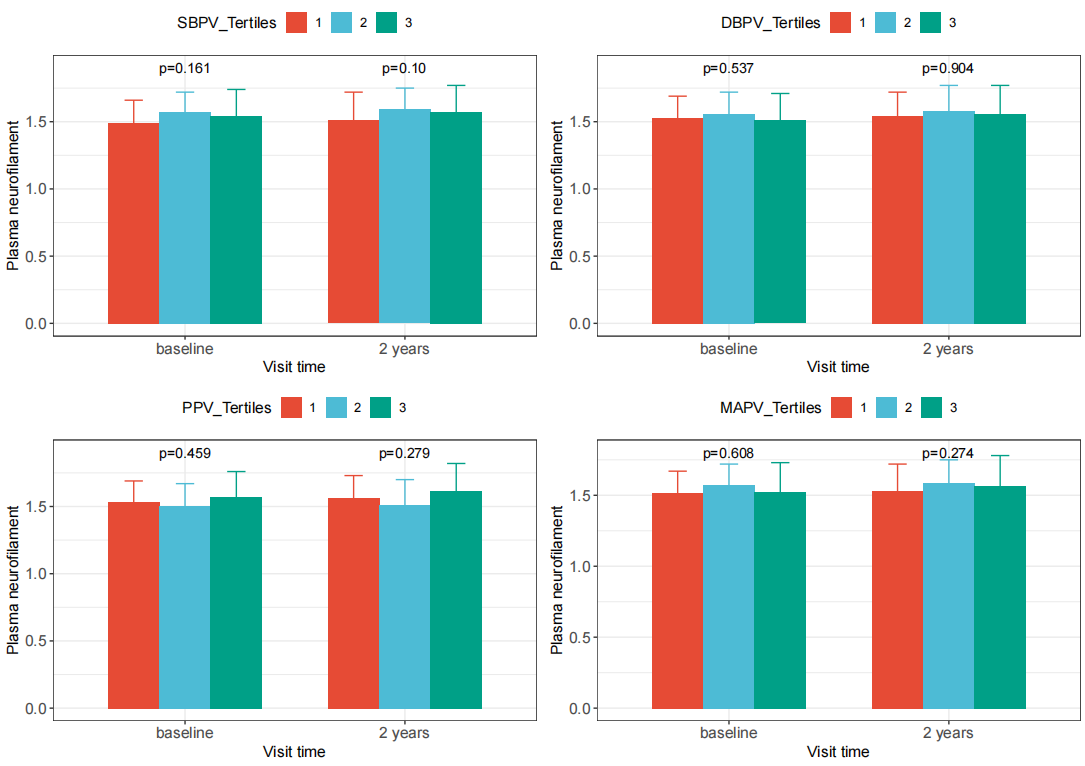


Note: BPV tertiles, level 1 represented the low tertile, level 2 represented the medium tertile, and level 3 represented the high tertile. SBPV, systolic blood pressure variability; DBPV, diastolic blood pressure; PPV, pulse pressure variability; MAPV, mean arterial pressure variability.

Figure S7 Plasma neurofilament light Levels at baseline and 2 years among different blood pressure variability groups (by Tertile) in Aβ+WMH+ group


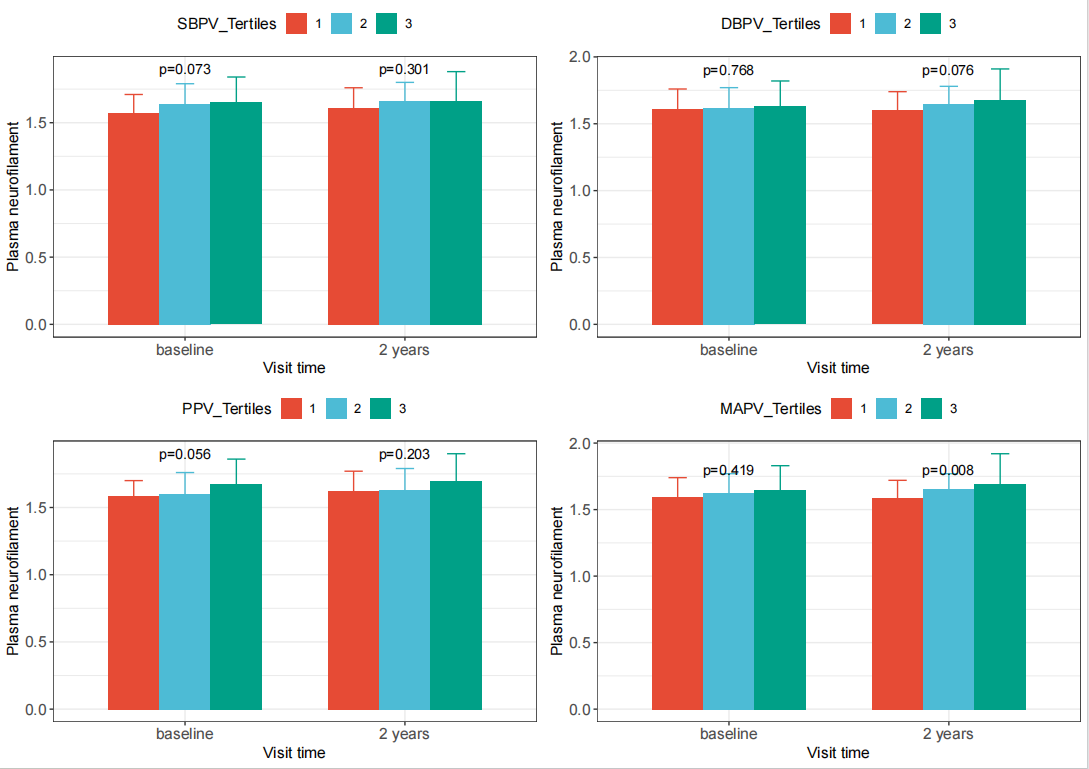


Note: BPV tertiles, level 1 represented the low tertile, level 2 represented the medium tertile, and level 3 represented the high tertile. SBPV, systolic blood pressure variability; DBPV, diastolic blood pressure; PPV, pulse pressure variability; MAPV, mean arterial pressure variability.

Figure S8 Plasma neurofilament light Levels at baseline and 2 years among different blood pressure variability groups (by Tertile) in Aβ-positive individuals


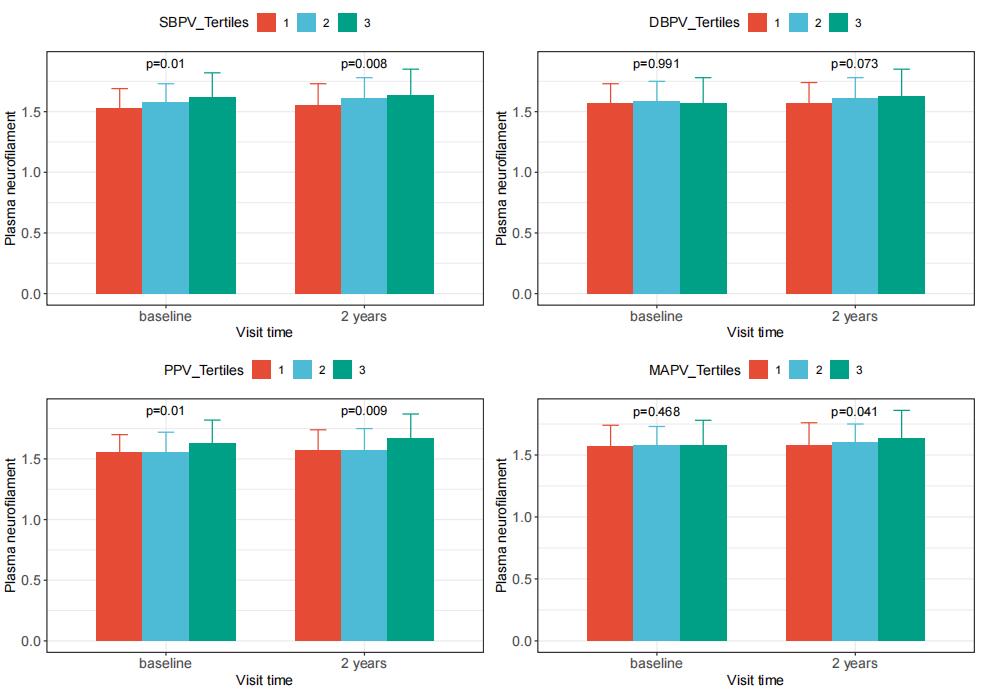


Note: BPV tertiles, level 1 represented the low tertile, level 2 represented the medium tertile, and level 3 represented the high tertile. SBPV, systolic blood pressure variability; DBPV, diastolic blood pressure; PPV, pulse pressure variability; MAPV, mean arterial pressure variability.

Figure S9 Plasma neurofilament light Levels at baseline and 2 years among different blood pressure variability groups (by Tertile) in Aβ-negative individuals


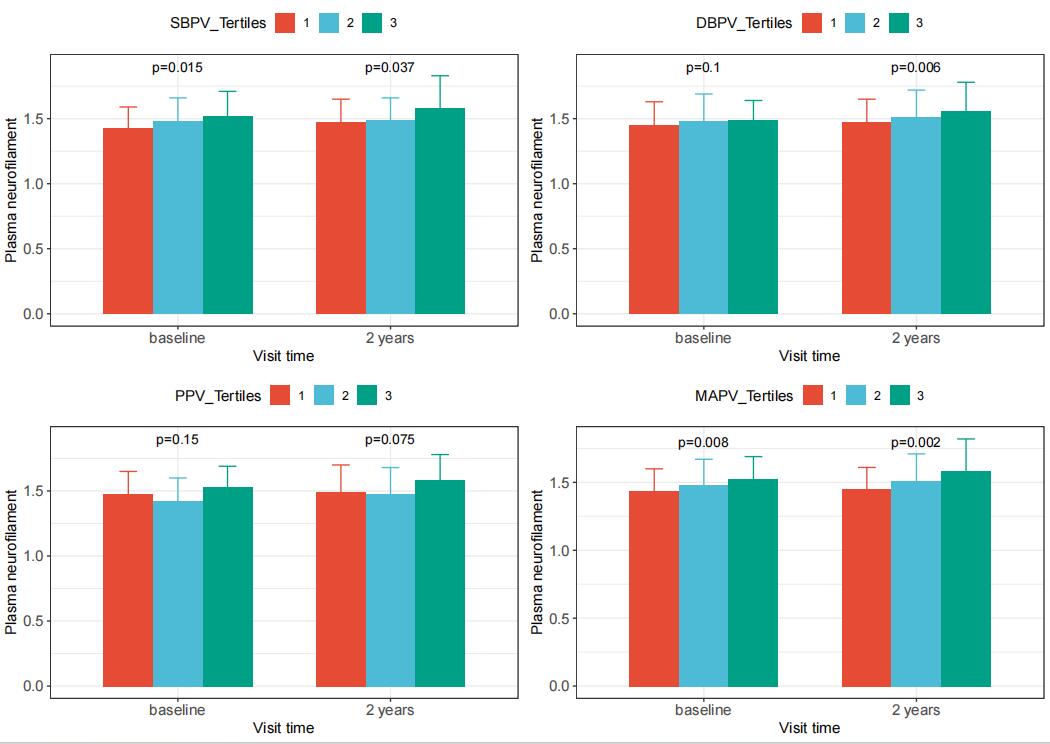


Note: BPV tertiles, level 1 represented the low tertile, level 2 represented the medium tertile, and level 3 represented the high tertile. SBPV, systolic blood pressure variability; DBPV, diastolic blood pressure; PPV, pulse pressure variability; MAPV, mean arterial pressure variability.

Figure S10 The mediation effect of plasma neurofilament light on the association of blood pressure variability with brain structural changes and cognitive performance at 2 years in the Aβ-WMH- group


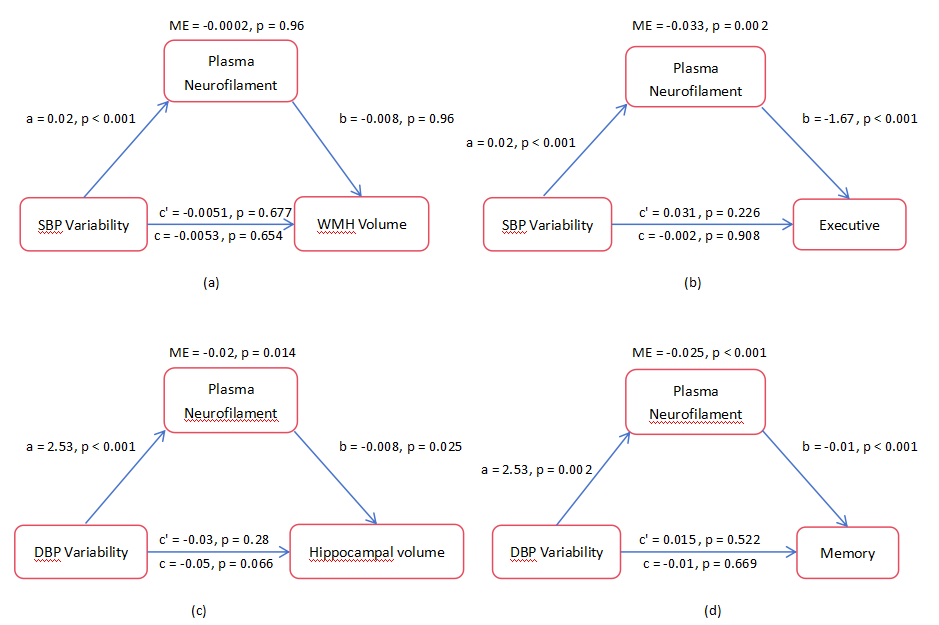


Abbreviation: WMH, white matter hyperintensities; ME, mediation effect; a, the effect of blood pressure variability on the mediator variable; b, the effect of the mediator variable on the outcome variable; c', direct effect; c, total effect.

Figure S11 The mediation effect of plasma neurofilament light on the association of blood pressure variability with brain structural changes and cognitive performance at 2 years in the Aβ-WMH+ group


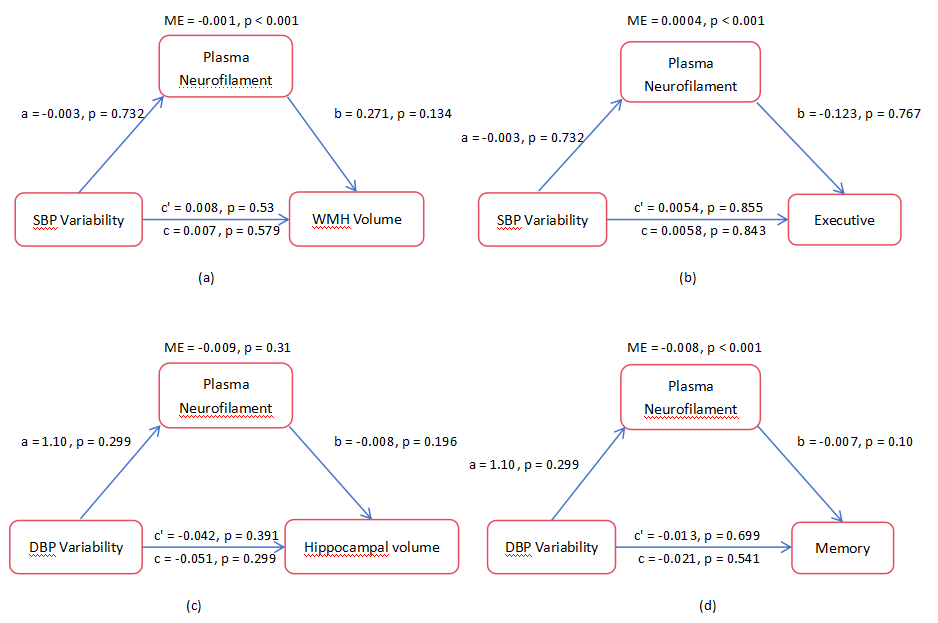


Abbreviation: WMH, white matter hyperintensities; ME, mediation effect; a, the effect of blood pressure variability on the mediator variable; b, the effect of the mediator variable on the outcome variable; c', direct effect; c, total effect.

Figure S12 The mediation effect of plasma neurofilament light on the association of blood pressure variability with brain structural changes and cognitive performance at 2 years in the Aβ+WMH- group


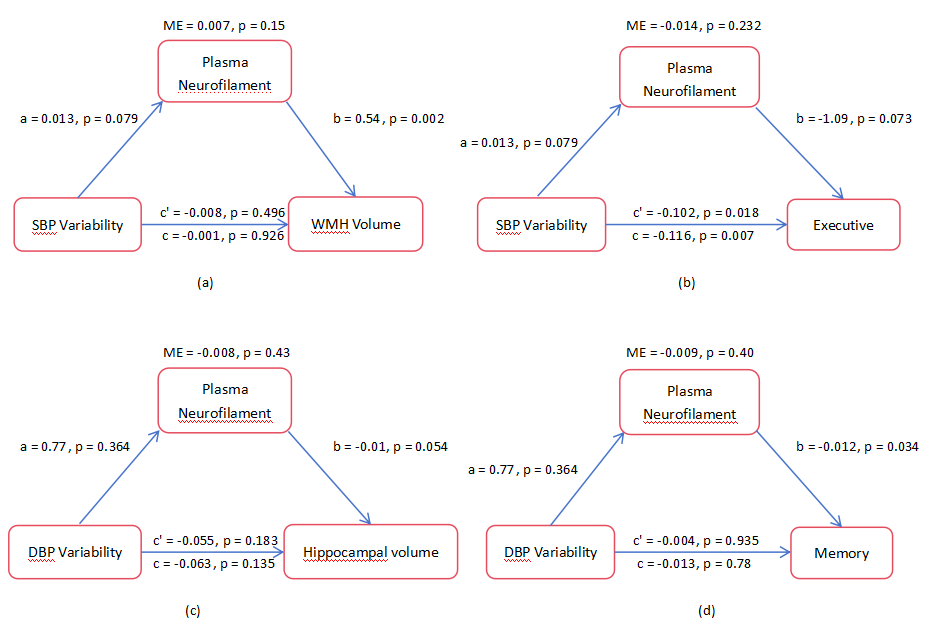


Abbreviation: WMH, white matter hyperintensities; ME, mediation effect; a, the effect of blood pressure variability on the mediator variable; b, the effect of the mediator variable on the outcome variable; c', direct effect; c, total effect.

Figure S13 The mediation effect of plasma neurofilament light on the association of blood pressure variability with brain structural changes and cognitive performance at 2 years in the Aβ+WMH+ group


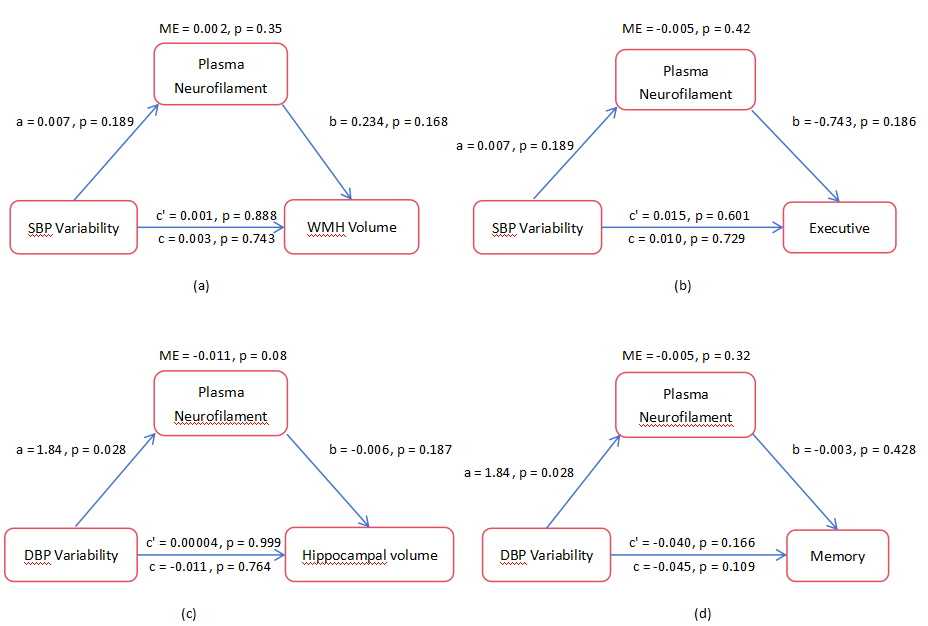


Abbreviation: WMH, white matter hyperintensities; ME, mediation effect; a, the effect of blood pressure variability on the mediator variable; b, the effect of the mediator variable on the outcome variable; c', direct effect; c, total effect.

Figure S14 The mediation effect of plasma neurofilament light on the association of blood pressure variability with brain structural changes and cognitive performance at 2 years in Aβ-positive individuals


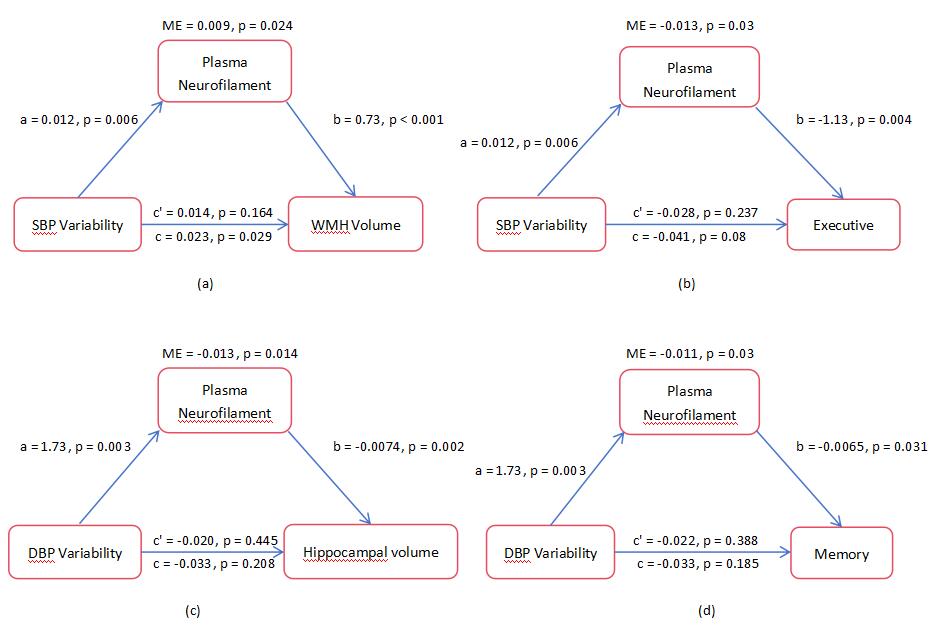


Abbreviation: WMH, white matter hyperintensities; ME, mediation effect; a, the effect of blood pressure variability on the mediator variable; b, the effect of the mediator variable on the outcome variable; c', direct effect; c, total effect.

Figure S15 The mediation effect of plasma neurofilament light on the association of blood pressure variability with brain structural changes and cognitive performance at 2 years in Aβ-negative individuals


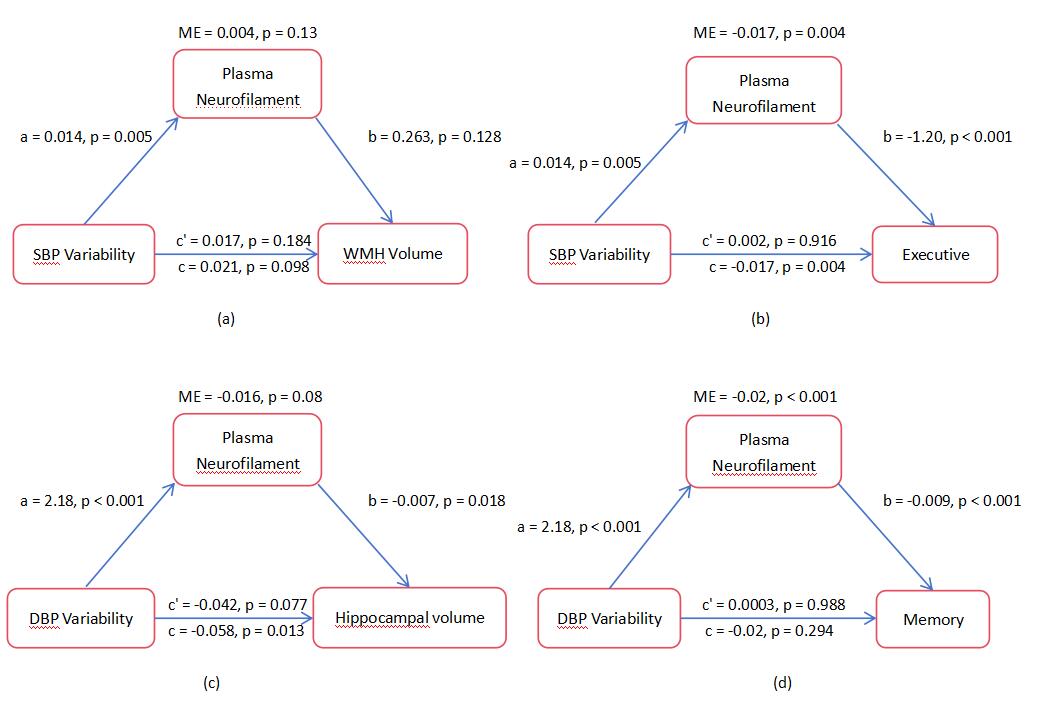


Abbreviation: WMH, white matter hyperintensities; ME, mediation effect; a, the effect of blood pressure variability on the mediator variable; b, the effect of the mediator variable on the outcome variable; c', direct effect; c, total effect.
